# The Biotechnological Applications of Marine Bacteria

**DOI:** 10.3390/biology15171546

**Published:** 2026-09-04

**Authors:** Liren Jiang, Robyn Wright, Renee Raudonis, Arjun H. Banskota, Bernard R. Glick, Robert J. Mitchell, Michal Scur, Zui Wang, Wenting Zhang, Tengfei Zhang, Qingping Luo, Morgan G. I. Langille, Zhenyu Cheng, Guoyuan Wen

**Affiliations:** 1 Key Laboratory of Prevention and Control Agents for Animal Bacteriosis, Ministry of Agriculture and Rural Affairs, Hubei Provincial Key Laboratory of Animal Pathogenic Microbiology, Institute of Animal Husbandry and Veterinary, Hubei Academy of Agricultural Sciences, Wuhan 430061, China; vd540170@dal.ca (L.J.); cr675792@dal.ca (Z.W.); wentingzhang87@hotmail.com (W.Z.); tfzhang23@163.com (T.Z.); qingping0523@163.com (Q.L.); 2 Department of Microbiology and Immunology, Faculty of Medicine, Dalhousie University, Halifax, NS B3H 4R2, Canada; r.raudonis@dal.ca (R.R.); michal.scur@gmail.com (M.S.); 3 Department of Pharmacology, Faculty of Medicine, Dalhousie University, Halifax, NS B3H 4R2, Canada; robyn.wright@dal.ca (R.W.); morgan.g.i.langille@dal.ca (M.G.I.L.); 4 Aquatic and Crop Resource Development Research Centre, National Research Council Canada, Halifax, NS B3H 3Z1, Canada; arjun.banskota@nrc-cnrc.gc.ca; 5 Department of Biology, University of Waterloo, Waterloo, ON N2L 3G1, Canada; glick.bernard@gmail.com; 6 Applied Microbiology Lab, School of Life Sciences, Ulsan National Institute of Science and Technology, Ulsan 44919, Republic of Korea; esgott@unist.ac.kr

**Keywords:** marine bacteria, environment cleanup, microbiome, analytical chemistry, biocontrol, nutraceuticals, antibacterial

## Abstract

Marine bacteria represent a vast and largely untapped resource for biotechnological innovation, offering solutions to global challenges in health, sustainability, and environmental conservation. The ocean’s unique conditions have driven marine bacteria to evolve diverse metabolic capabilities, resulting in the production of bioactive compounds, enzymes, and other metabolites with wide-ranging applications. Recent advances in high-throughput sequencing, metagenomics, and analytical chemistry have unlocked new opportunities for leveraging these microorganisms in fields as varied as medicine, agriculture, and bioremediation. This review highlights the role of marine bacteria in the One Health framework, showcasing their contributions to antimicrobial discovery, nutraceutical development, pathogen biocontrol, and environmental cleanup, including microplastic degradation. This review also examines emerging methodologies such as microbiome mining and advanced culturing techniques, which hold the key to realizing the full potential of marine bacteria in a sustainable bioeconomy. By bridging fundamental research with applied sciences, marine biotechnology promises to deliver transformative impacts on human, animal, and environmental health.

## Simple Summary

The world’s oceans are filled with tiny, invisible bacteria that could help solve major problems facing people, animals, and the environment. Scientists are studying these marine bacteria because they produce natural chemicals and tools that we can use. For example, some of these bacteria may lead to the development of new antibiotics to fight infections, while others could help create healthy food supplements or control diseases in farmed fish without harsh chemicals. Marine bacteria can also help clean up pollution, including breaking down harmful microplastics in the water. New technologies, such as advanced DNA sequencing and special growing methods, now allow researchers to discover and use these bacterial abilities more easily than ever before. By learning from the sea’s smallest organisms, we can develop better medicines, protect our food supply, and restore a cleaner, healthier planet. This research connects what happens in nature directly to benefits for society.

## 1. Introduction

We are in the midst of the Decade of Ocean Science for Sustainable Development (2021 to 2030) proclaimed at the 2017 United Nations (UN) General Assembly. The purpose of the UN-declared ‘Ocean Decade’ (https://www.unesco.org/en/decades/ocean-decade, accessed on 27 January 2025) is “to stimulate ocean science and knowledge generation to reverse the decline of the state of the ocean system and catalyze new opportunities for sustainable development of this massive marine ecosystem”. The ocean-based bioeconomy is recognized as a significant contributor to global economic growth [1,2,3,4,5]. Central to this bioeconomy is the sustainable utilization of marine bioresources, among which marine bacteria represent one of the most promising yet underutilized assets. These microorganisms, through their vast genetic and metabolic diversity, offer transformative solutions for developing novel pharmaceuticals, biodegradable materials, and environmentally friendly industrial processes. On the other hand, anthropologic effects, such as global population increase and climate change, underlie a pressing need for technological innovations in responsible bioeconomy to address the increasing demands for the health of human beings and our surrounding environments. Marine biotechnology holds tremendous potential in transforming the diversity of ocean biomass and resources into innovative and sustainable aquaculture, energy, biomaterials, medicine, environment cleanup, and other applications [6].

It was estimated that the total bacterial biomass in the ocean was equivalent to approximately 2 billion tonnes of carbon, representing nearly a quarter of the living biomass in the oceans [7,8]. The diverse marine environmental conditions in terms of temperature, salinity, shear pressure and light levels drive the immense metabolic and functional diversity in marine bacteria, which make fundamental contributions to maintaining a healthy biogeochemical homeostasis within marine ecosystems [9,10], but are also implemented in numerous applications in agriculture, industry, and medicine [6]. This functional diversity is closely linked to biogeography: marine bacteria from different regions produce distinct metabolites shaped by local environmental pressures. For example, polar species are rich sources of cold-active enzymes, while tropical and deep-sea bacteria often yield structurally complex bioactive compounds with pharmaceutical potential [11]. The exploration of marine bacterial biodiversity has been revolutionized by the development of culture-independent high-throughput sequencing technology [12,13,14], which in turn enabled the discovery potential of new organisms, their products and related biosynthetic pathways for biotechnological advances [15,16,17].

These bacteria exhibit a wide range of evolutionary traits, producing diverse bioactive substances such as secondary metabolites, enzymes, and biopolymers [18,19,20,21]. Many marine bacteria and their metabolites have been identified and utilized in agriculture, industry, and medicine [22,23,24,25,26]. A notable example of marine bacterial biotechnology is the contribution of natural products to modern medicine. While over 50% of current medicines are derived from natural compounds or their synthetic derivatives, primarily from terrestrial plants, fungi, and soil bacteria, marine bacteria have emerged as a promising and underexplored source of novel bioactive molecules. This percentage is even higher for anticancer and antimicrobial treatments. For example, salinosporamide A (marizomib), a highly potent proteasome inhibitor, was initially identified in the marine bacterium *Salinispora tropica* and has been investigated for treating multiple forms of cancer [27,28,29].

In addition to the direct use of marine bacterial metabolites in various applications such as consumable drugs, marine organisms can benefit various industries and medical fields through their enzymatic activities or whole bacterial cells. For instance, marine bacteria or their enzymes are effective in removing and degrading chemical compounds or organic matter, which can be upcycled into value-added products or used for environmental clean-up. Additionally, marine bacteria can serve as biocontrol agents in agriculture and aquaculture, targeting bacterial pathogens to control infectious diseases and reduce antibiotic use.

Despite these opportunities, the application of marine bacteria in biotechnology is constrained by major challenges, including the poor culturability of most marine species, low yields of bioactive compounds, and difficulties in genetic manipulation [6]. Recent advances in biotechnology, societal engagement, and global partnerships are paving the way for marine bacterial biotechnology to benefit the bioeconomy, environment, and human society. This review aims to synthesize recent advances in the practical applications of marine bacteria and their products within the framework of One Health, highlighting selected examples of how they promote the health of plants, animals, humans, and the environment. We also focus on methodologies, including bacterial cultivation, analytical chemistry, and microbiome mining, which will facilitate the realization of the full potential of these applications.

## 2. Methods

As a narrative review covering a broad multidisciplinary field, this work was prepared through a collaborative expert-driven approach. Each co-author performed targeted literature searches in their area of expertise using PubMed, Web of Science, and Google Scholar, focusing on peer-reviewed articles published primarily between 2013 and 2024. Key search terms included combinations of “marine bacteria”, “biotechnology”, “antimicrobial”, “biocontrol”, “nutraceuticals”, “bioremediation”, “microplastics”, and “cultivation methods”. The final manuscript was cross-reviewed by all authors to ensure comprehensive coverage and accuracy.

## 3. Results

### 3.1. Marine Bacteria Applications in One Health

**Figure 1:**
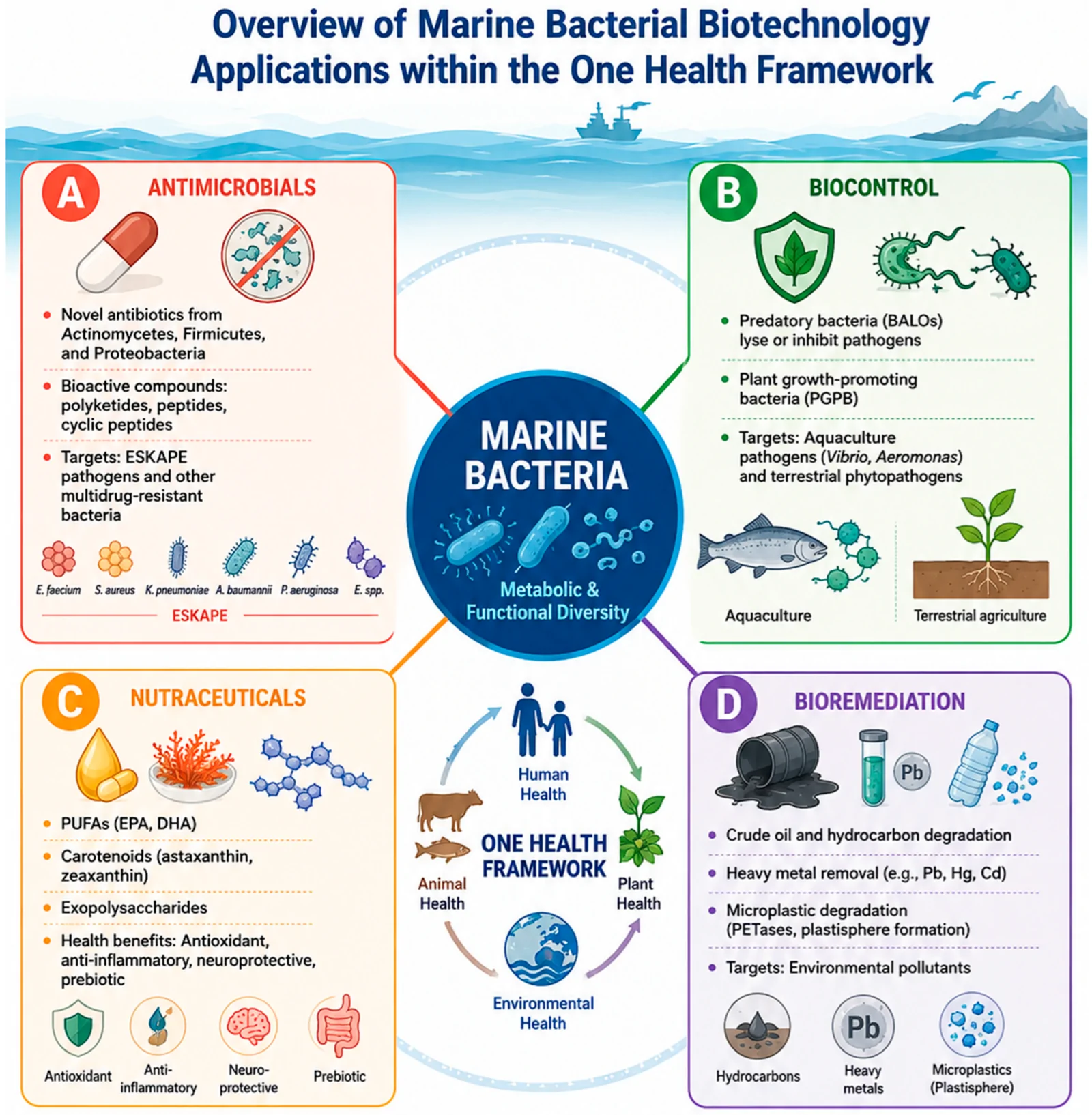
Overview of marine bacterial biotechnology applications within the One Health framework. Marine bacteria serve as a central biotechnological resource for four major application areas, each contributing to interconnected health outcomes. (**A**) Antimicrobials: marine bacteria produce diverse secondary metabolites—including polyketides, peptides, and cyclic peptides—with activity against drug-resistant ESKAPE pathogens and other clinically relevant bacteria. (**B**) Biocontrol: predatory bacteria (BALOs) and plant growth-promoting marine bacteria suppress pathogens in aquaculture and terrestrial agriculture, reducing reliance on chemical pesticides and antibiotics. (**C**) Nutraceuticals: marine bacteria synthesize health-promoting compounds such as PUFAs (EPA, DHA), carotenoids (astaxanthin, zeaxanthin), and exopolysaccharides with antioxidant, anti-inflammatory, neuroprotective, and prebiotic properties. (**D**) Bioremediation: marine bacteria degrade environmental pollutants including crude oil, heavy metals, and microplastics through enzymatic pathways and community-level processes such as plastisphere formation.

The applications of marine bacteria in various fields have been extensively reviewed. In this work, we focus on summarizing recent advances with an emphasis on the integrated One Health framework, addressing the health of the environment and living organisms. This includes emerging concerns such as microplastic contamination, as well as traditional applications like nutritional benefits and antimicrobial medicine. We highlight their roles in combating pathogens across a wide range of hosts, from plants to humans. Additionally, we distinguish between antimicrobial mechanisms driven by microbe-derived metabolites and those involving whole bacteria in biocontrol, with a unique focus on predatory bacteria as a case study for pathogen control (Figure 1).

#### 3.1.1. Nutraceuticals

Nutraceuticals are products derived from food and non-food sources and are dietary supplements marketed as providing health benefits beyond basic nutrition. There is increased interest in these products that possess medicinal or health-promoting properties, as there is a growing market for natural ways to promote health [30,31,32,33,34,35,36]. Numerous marine organisms are already being used in the production of nutraceuticals, and marine bacteria are emerging as valuable contributors, as they offer unique and diverse enzymatic capabilities and bioactive compounds.

Marine bacterial species have shown potential for nutraceutical production, including members of genera such as *Alteromonas*, *Bacillus*, *Marinobacter*, *Pseudoalteromonas*, *Streptomyces*, and *Vibrio*, among others. These marine bacteria produce a range of bioactive compounds, including secondary metabolites, polysaccharides, pigments, and peptides that can benefit human and animal health [37,38,39]. Table 1 summarizes some nutraceutical candidates reported from marine bacteria. For a deeper view of the diversity and nutraceutical potential of marine bacterial species and their bioproducts, see the review by Maldonado-Ruiz et al. [39].

The impact of these marine-derived nutraceuticals on the host microbiome and overall health is an area of growing interest. Nutrients and bioactive compounds provided by marine bacteria, such as essential fatty acids and antioxidants, contribute to overall health and well-being by supporting metabolic processes and reducing oxidative stress [36,37,38,39]. One example of a marine-derived bacterial compound with antioxidant capability is NP7 from *Streptomyces* sp., which can cross the blood–brain barrier and act as a neuroprotective agent, as it prevents apoptosis, necrosis and an increase in the phosphorylation of ERK (extracellular signal-regulated kinase) caused by H_2_O_2_ [58,59]. Marine bacterial exopolysaccharides (EPSs) are particularly versatile; their physicochemical properties, including metal-chelating, emulsifying, and immunomodulatory activities, underpin applications ranging from wastewater treatment and cosmetics to cancer therapy [39]. EPSs can also be used as probiotics for maintaining intestinal health, as studies in mice with purified EPSs from *Gelidium pacificum* and *Cereus sinensis* resulted in reduced intestinal inflammation and a change in the gut microbiota composition, increasing beneficial bacteria [60].

In addition to their role in producing bioactive compounds for aiding human health, marine bacteria are also useful for supplementing animal and plant health. Marine bacteria have the potential to be used as probiotics in agriculture and aquaculture [61,62]. Additionally, they are a source of various enzymes that are instrumental in breaking down complex compounds into simpler, more digestible forms, improving their nutritional value and digestibility. For example, enzymes produced by marine bacteria can degrade algal alginate and fucoidan, two significant polysaccharides found in brown algae that have potential applications in aquaculture and agriculture. Alginate and fucoidan are valuable in animal feeds for their probiotic properties, which can enhance the growth and health of aquaculture species and livestock [63,64,65,66,67,68]. These enzymes also reduce waste production, as they decrease the amount of undigested algal materials used in the aquaculture industry [64,69]. Additionally, some studies suggest that the breakdown products of alginate and fucoidan might have plant growth-promoting effects when used in composts or as soil additives, as they can stimulate beneficial soil microorganisms, enhancing soil health and fertility [70,71].

In summary, marine bacteria are a promising source of bioactive compounds and enzymes for nutraceutical production. Their ability to produce essential nutrients, degrade complex polysaccharides, and contribute to a healthy host microbiome enhances their utility in health and nutrition applications. As research advances, the role of marine bacteria in developing nutraceuticals is likely to expand, providing new opportunities for enhancing health.

#### 3.1.2. Biocontrol

The yield of terrestrial plants is often reduced by ~10% (in developed countries) to ~20% (in less developed countries) by plant diseases largely caused by various phytopathogenic fungi and bacteria [72]. Most of the known phytopathogenic fungi belong to the Ascomycetes such as *Fusarium* spp., *Thielaviopsis* spp., *Verticillium* spp., *Magnaporthe grisea* and *Sclerotinia sclerotiorum* and the Basidiomycetes such as *Ustilago* spp., *Rhizoctonia* spp., *Phakospora pachyrhizi* and *Puccinia* spp. [72].

Like terrestrial plants (and animals), many marine plants and animals are also subject to severe growth inhibition by various pathogenic marine bacteria and fungi [73,74,75,76]. In addition, since they can often aggressively degrade lignocellulose, many marine fungi are common degraders of dead animals and plants in marine environments [77].

Farmers have traditionally used chemical pesticides to control the proliferation of terrestrial phytopathogens and thereby limit the damage that they cause to crop plants [70]. However, many chemical pesticides produce unwanted side effects and are often toxic to both animals and humans [78]. In humans, some of these pesticides can cause headache, nausea, vomiting, fatigue, and irritation of skin and eyes. Relatively recently (i.e., within the past ~25 years), scientists have isolated and successfully tested several terrestrial biocontrol plant growth-promoting bacteria (PGPB) as safe, efficacious and natural means of controlling the functioning and proliferation of phytopathogens [79]. While there are as yet only a limited number of reports of the isolation and characterization of plant growth-promoting microbes (PGPMs) from marine environments, this is a promising and growing endeavor (Table 2). In the past ~20 years, several marine PGPMs have been isolated, identified, and preliminarily characterized. However, it should be noted that in many of the instances mentioned in Table 2, the isolated biocontrol strain was not characterized to both the genus and species level, and the putative marine biocontrol strain(s) was often not tested under marine conditions. In addition, while some plant growth-promoting and biocontrol mechanisms have been identified in a portion of these marine biocontrol strains, the information that has been reported to date is generally insufficient to specifically delineate the precise biocontrol mechanism(s) that are employed by these organisms in a natural aqueous marine setting. Nevertheless, the inclusion of these studies is justified as they demonstrate the broader potential of marine bacteria to address agricultural challenges beyond the marine environment, which is a key translational aspect of marine biotechnology.

Beside controlling bacterial pathogens causing diseases in terrestrial plants, some marine bacteria can also target several pathogens that pose significant threats to the health of marine organisms, including fish, crustaceans, mollusks, and even corals (Table 2). These pathogens include *Vibrio*, *Aeromonas*, *Pseudomonas*, and *Flavobacterium* species, which are notorious for causing devastating diseases in aquaculture and wild marine populations. For instance, *Vibrio* species, in particular, *V. parahaemolyticus* [97,98], *V. anguillarum* [99,100], *V. vulnificus* [101,102] and *V. harveyi* [98,103], are among the most virulent pathogens in marine systems, causing diseases like acute hepatopancreatic necrosis disease (AHPND) [98] and vibriosis [104] in marine life. Several of these pathogens are also zoonotic and quite capable of infecting humans, either through the consumption of raw or undercooked seafood or through skin wounds [105,106,107,108]. Several other major marine pathogens include *Aeromonas* spp., especially *A. hydrophila* and *A. salmonicida*, which cause furunculosis and hemorrhagic septicemia in various fish species [109,110], leading to significant economic losses in aquaculture [111,112].

**Figure 2:**
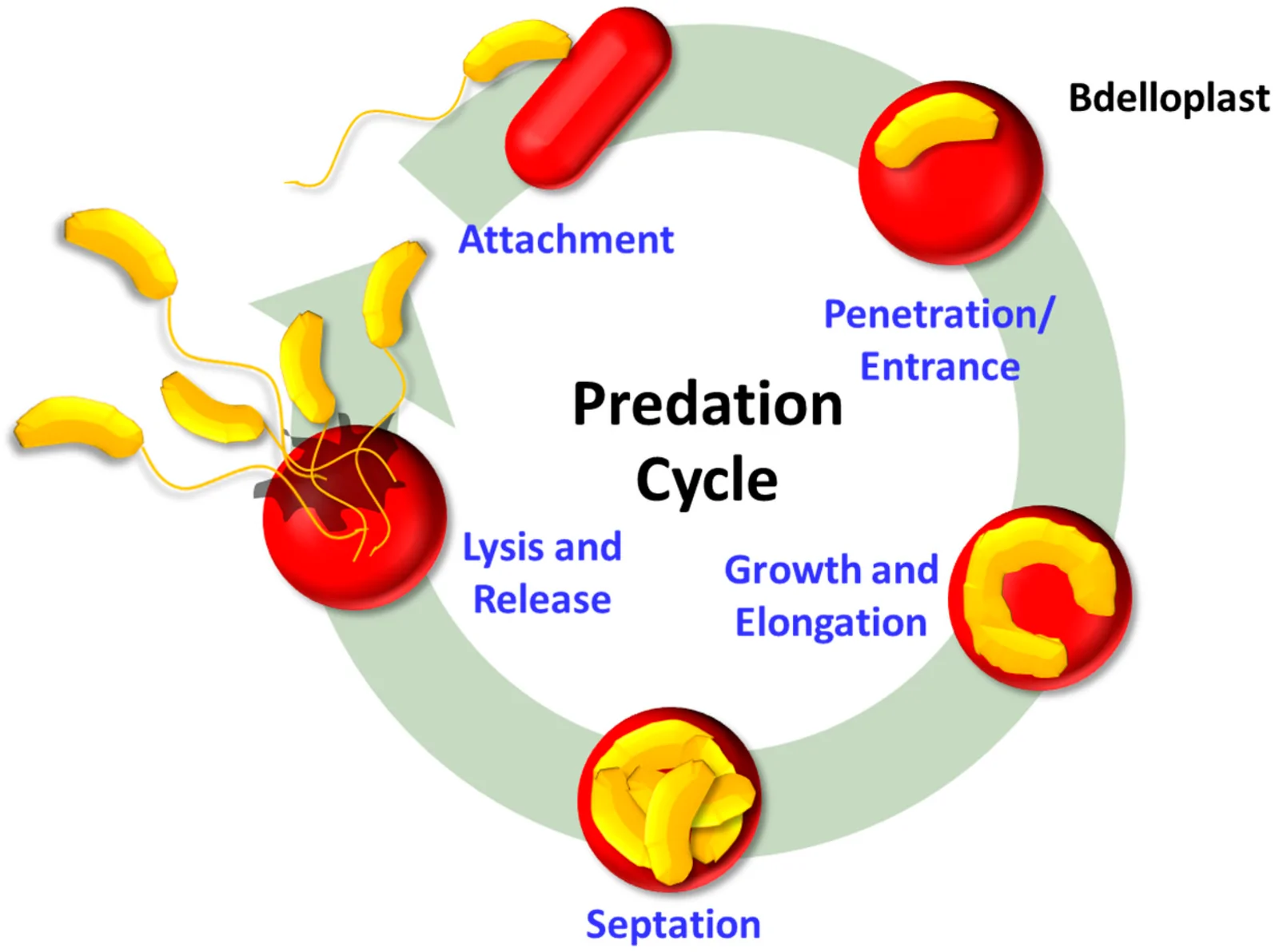
The predation cycle of intraperiplasmic BALO bacterial strains. Shortly after attachment of a BALO (yellow) to a susceptible prey (red), the predator enters the periplasmic space and establishes itself, forming the bdelloplast. Here, the predator continues to hydrolyze and consume the prey cell components, supporting its own growth. As the prey nutrients are exhausted, the predator undergoes septation and lyses the prey, releasing daughter predators to attack other prey.

While many marine bacteria exert biocontrol through antibiotic production or competition, these mechanisms are often constrained by resistance development and limited host range. Predatory bacteria, which actively hunt and consume other bacteria, offer a distinct and potentially more robust alternative (Table 3). These microbial predators represent a unique and fascinating option for biocontrol, characterized by their abilities to attack, kill, and consume other bacteria as a source of nutrients and energy (Figure 2) [113]. Among the most studied predatory bacteria are the *Bdellovibrio*-and-like organisms (BALOs), which include genera such as *Bdellovibrio*, *Bacteriovorax*, and *Halobacteriovorax* [113,114,115]. All these microbes are Gram-negative, obligate aerobes that exhibit a bi-phasic lifestyle, consisting of a free-swimming attack phase and an intraperiplasmic growth phase within their prey [116], although this idea has been challenged recently [117]. Not only are these microbes present across the globe, from pole to pole and in-between [118,119,120], BALOs exhibit a range of predatory behaviors and possess specialized adaptations that facilitate their predatory lifestyle [121,122,123], which plays a crucial role in shaping microbial communities in their natural environments [124,125].

The benefits of using predatory bacteria as biocontrol measures in marine environments have primarily revolved around their ability to significantly reduce the populations of the pathogens listed in Table 3, thereby reducing the incidence and severity of infectious diseases in marine animals [126,127,128,129]. This natural biocontrol mechanism is particularly useful in aquaculture settings where high stocking densities can exacerbate the spread of diseases. A number of studies also explored the safety of predatory bacteria towards higher organisms and found they have little to no toxicity, whether towards cells [130,131] or the health of whole organisms [128,132,133,134].

**Table 3:** Some major marine bacterial pathogens and their susceptibility to predation by BALOs.

Pathogen	Examples of Aquatic Animals Infected	Predation *	References
*Aeromonas* spp.	Rainbow Trout, Catfish, Shrimp	+++	[135,136,137,138]
*Vibrio anguillarum*	Sea Mullet, Salmon, Turbot	++	[139]
*Vibrio harveyi*	Barramundi, Flounder, Shrimp	n.d.	[140,141]
*Vibrio parahaemolyticus*	Clownfish, Shrimp	+++	[142,143]
*Vibrio vulnificus*	European Eel, Shrimp	+++	[144]

* Predation activity: ++, good activity (approx. 1- to 2-log units reduction); +++, strong activity (>3-log units reduction); n.d., not determined.

A second benefit offered by BALOs is they may also help to restore and maintain the balance of microbial communities in marine systems. By selectively targeting pathogenic bacteria, they can reduce the competitive pressure on beneficial microbes, allowing for a more diverse and stable environmental microbiome, even within the guts of marine animals, where greater richness (i.e., diversity) and abundance of predators positively correlates with gut diversity [124]. This enhanced microbial diversity is known to contribute towards better overall ecosystem health and resilience against disease outbreaks [145].

Finally, BALOs not only kill pathogens, but recent works have also highlighted their propensities to effectively remove antibiotic-resistant genes (ARGs) that are present in their prey [146,147], with current evidence pointing towards the activities of two nucleases encoded by the predator [148]. In the former studies, predation reduced the ARGs by 2- to 3-log units, results that were commensurate with their killing efficiencies against the pathogens, while one of these studies also found that the number of functional plasmids also diminished [147]. As such, BALOs represent a natural and environmentally friendly alternative to traditional chemical treatments and antibiotics, which are often associated with the development of antibiotic-resistant strains and environmental contamination, and their use aligns with the principles of sustainable aquaculture and supports the production of safer and healthier seafood products.

#### 3.1.3. Antimicrobials

Marine bacteria, adapting to competitive pressures in challenging environments, deploy a range of strategies and specialized metabolic mechanisms to thrive. In addition to the biocontrol and predatory tactics discussed above, these bacteria are notable for producing a variety of antimicrobial compounds. While the antibacterial compounds from marine bacteria have been reviewed [149,150,151,152], this section briefly summarizes recent studies on antibacterial agents derived from marine bacteria, focusing on their unique characteristics that have garnered attention in biotechnology.

The marine environment harbors antimicrobial producers across multiple bacterial phyla, each with distinct ecological niches and chemical profiles. Actinomycetes, particularly *Streptomyces* species, are the most extensively studied and prolific producers, dominating marine sediment communities and yielding diverse secondary metabolites such as polyketides, peptides, and glycosides [153]. Their prevalence in discovery efforts reflects both their metabolic richness and the relative ease of cultivation from marine sediments. For example, Sabido et al. [154] demonstrated that *Streptomyces* isolates from marine sediments produce crude extracts with varying levels of inhibition against ESKAPE pathogens, which are particularly dangerous for immunocompromised individuals [154,155].

Firmicutes, especially *Bacillus* species, represent a second major source of marine antimicrobials. Unlike Actinomycetes, which often produce specialized metabolites through large biosynthetic gene clusters, *Bacillus* spp. are known for producing ribosomally synthesized and post-translationally modified peptides (RiPPs), as well as polyketides with broad-spectrum activity. For instance, Kizhakkekalam and Chakraborty [156] identified a polyketide from *Bacillus amyloliquefaciens* with activity against drug-resistant *Staphylococcus aureus* and *Pseudomonas aeruginosa*. Notably, Firmicutes-derived compounds frequently exhibit broader spectrum activity compared to many Actinomycete metabolites, effectively targeting multiple resistant strains [156].

Proteobacteria, encompassing classes such as Gammaproteobacteria, remain comparatively underexplored as a source of marine antimicrobials [157]. This is partly due to cultivation challenges, as many marine Proteobacteria require specific growth conditions, and partly due to concerns regarding their pathogenicity and complex symbiotic relationships with eukaryotic hosts. However, emerging evidence suggests that Proteobacteria produce structurally unique compounds, including macrocyclic polyketides from *Shewanella* species and other metabolites from *Vibrio* and *Pseudomonas* genera. The underrepresentation of Proteobacteria in current discovery pipelines likely reflects sampling biases and technical difficulties rather than an actual lack of biosynthetic potential.

As shown in Table 4, marine bacteria produce a diverse array of secondary metabolites with antimicrobial effects against resistant pathogens. Several trends emerge from this compilation: (1) Actinomycetes dominate the reported discoveries, reflecting historical focus and methodological advantages; (2) Firmicutes, while less frequently reported, produce compounds with notably broad-spectrum activity against multiple resistant strains; and (3) Proteobacteria-derived compounds are rare but structurally distinct. Regarding specific compound classes, while resistance to traditional β-lactams is common, novel β-lactams and other scaffolds isolated from marine sources may offer new approaches to overcoming resistance through broader spectrum activity and unique structural features. However, many of these compounds are difficult to extract or produce at scale, necessitating improved cultivation, fermentation, and characterization techniques.

Marine bacteria are also a rich source of antibacterial peptides, as well-documented by Desriac et al. [158], with cyclic peptides from marine sources, including marine bacteria, reviewed by Ribeiro et al. [159]. The comparative analysis of antibacterial metabolites across these phyla highlights the need for balanced discovery efforts, including improved cultivation strategies for underrepresented groups, to fully harness marine bacterial diversity as a resource against drug-resistant pathogens.

**Table 4:** Antimicrobial activity of different marine bacteria.

Bacterial Species	Extract/ Compounds	Target Pathogens	References
*Streptomyces* sp. MBTG32	Tunicamycin VII, VIII, IX Corynetoxin U17a	*S. aureus*	[160]
*Streptomyces olivaceus* SCSIO 1071 ΔdtxR	Olenamidonins A-C	*E. faecalis*, *E. faecium*	[161]
*Streptomyces* sp. NBU3429	Xiamycin Chlorinated metabolite Chloroxiamycin	*S. aureus*	[162]
*Micromonospora* CA-214671, CA-214658, CA-218877	Maklamicin, Maklamicin B, Phocoenamicin, Phocoenamicin B	*E. faecalis*, *E. faecium*, *M. tuberculosis*, *S. aureus*	[163]
*Streptomyces* A15ISP2-DRY2^T^	Antibiotic compounds	*E. faecalis*, *S. aureus*	[164]
*Streptomyces youssoufiensis* OUC6819 ΔdtlA	Youssoufenes A2, A3	*E. faecalis*	[165]
*Streptomyces parvus* SCSIO Mla-L010	Vicenistatin	*S. aureus*	[166]
*Streptomyces* sp. SBRK1	Kalafungin	*S. aureus*	[167]
*Nocardiopsis aegyptia* HDN19-252	Saliniquinones G-I; Heraclemycin E	Coagulase negative staphylococci	[168]
*Streptomyces scopuliridis* SCSIO ZJ46	Desotamides A4, A6	*S. aureus*	[169]
*Streptomyces* sp. MS110128	Cyclic peptides	*S. aureus*	[170]
*Streptomyces olivaceus* OUCLQ19-3	Dixiamycins	*E. faecalis*, *E. faecium*; *M. luteus*, *S. aureus*, *P. aeruginosa*	[171]
*Streptomyces costaricanus* SCSIO ZS0073	Actinomycin	*E. faecium*, *S. aureus*	[172]
*Streptomyces bacillaris* MBTC38	Lactoquinomycins	*S. aureus*	[173]
*Streptomyces* sp. MBTI36	Drivative Ap, A2, A3, A9	*S. aureus*	[174]
*Streptomyces atratus* SCSIO ZH16 ΔilaR mutant strain	Ilamycins G-R, E1, F	*M. tuberculosis*	[175]
*Streptomyces lusitanus* OUCT16-27	Angucycline	*E. faecalis*, *E. faecium*, *S. aureus*	[176]
*Bacillus zhangzhouensis SK4*	Phthalate	*P. aeruginosa*	[177]
*Heterotrophic Bacillus amyloliquefaciens* MTCC 12716	Amicoumacin-class of isocoumarin	*E. faecalis*, *K. pneumoniae*, *P. aeruginosa*, *S. aureus*, *S. flexneri*	[178]
*Bacillus amyloliquefaciens* MTCC12713	Difficidin	*E. faecalis*, *S. aureus*, *K. pneumoniae*, *P. aeruginosa*	[179]
*B. amyloliquefaciens MTCC 12716*	Macrolide	*E. faecalis*, *S. aureus*, *K. pneumoniae*, *P. aeruginosa*	[180]
*Bacillus atrophaeus* SHB2097	Polyketide Synthase; Nonribosomal Peptide Synthetase/Polyketide Synthase; Siderophore	*E. faecalis*, *S. aureus*, *K. pneumoniae*, *P. aeruginosa*	[181]
*Bacillus amyloliquefaciens* MBMS5	Ethyl acetate extract	*E. coli*, *S. aureus*, *V. parahemolyticus*	[182]
γ-proteobacterium *Shewanella*	Macrocyclic Polyketides	*E. faecalis*, *S. aureus*	[183]

#### 3.1.4. Bioremediation

Industrialization has released vast quantities of chemical pollutants into marine environments, including heavy metals (e.g., lead, cadmium, mercury), organic contaminants (e.g., petroleum hydrocarbons, pesticides, solvents), and emerging pollutants such as pharmaceuticals and plastics [184]. These pollutants enter water bodies through industrial discharge, agricultural runoff, and improper waste disposal, with an estimated 400 million tonnes reaching lakes, rivers, and ultimately the oceans each year [184]. Traditional physicochemical remediation methods, such as chemical precipitation, adsorption, and incineration, are often costly, energy-intensive, or generate secondary waste. In this context, biological approaches offer promising, eco-friendly alternatives [184].

Microalgae, including cyanobacteria and eukaryotic algae, contribute to bioremediation by removing inorganic pollutants (nitrogen, phosphates, and heavy metals) through biosorption and intracellular detoxification, while also degrading some organic contaminants [185,186,187,188,189,190,191,192,193,194]. However, the primary focus of this review is marine bacteria, which possess remarkable metabolic versatility for pollutant degradation. Marine bacteria facilitate the removal of a wide range of organic and inorganic toxicants [195,196,197].

Some of the prominent marine bacteria that have been shown to be involved in bioremediation include *Achromobacter*, *Acinetobacter*, *Alcanivorax*, *Alteromonas*, *Arthrobacter*, *Burkholderia*, *Bacillus*, *Cycloclasticus*, *Enterobacter*, *Flavobacterium*, *Marinobacter*, *Oleispira*, *Pseudomonas*, and *Thallassolituus* [198,199]. Table 5 summarizes some reports from the last 25 years on various marine bacteria that can (i) degrade organic contaminants and (ii) remove toxic metals from aqueous environments. In this regard, the major contaminant that has been addressed in these studies is crude oil and its components. While most of these reported studies have been performed under controlled laboratory conditions, there is every reason to believe that the isolated organisms could be used as effective adjuncts to help clean up the many crude oil spills that occur globally on a regular basis from both tankers and refineries.

#### 3.1.5. Emerging Contaminants

Another major public health concern is microplastic pollution, which has recently become a field of rigorous scientific study, as can be seen from the fact that most of the publications regarding this topic have been published in the past 5–7 years. Further impetus on microplastic research has been provided by the media giving attention to work identifying the presence of microplastics in various human tissues including the male reproductive tract [227,228], placenta [229], brain [230], and the blood stream and arteries [231,232]. Due to the ubiquitous presence of microplastics in the environment, and their accumulation in human and animal tissues, they have come under increasing scrutiny by regulatory agencies around the globe. Government and academic research groups have been steadily conducting studies to analyze what potentially harmful effects micro-and nano- plastic particles (MNPs) may have on the human body.

The main areas of this research are organs that come into immediate contact with MNPs such as lungs and the gastrointestinal tract, as well as the filter organs, which are the next likely destination for absorbed MNPs, such as the kidneys and liver [233]. Researchers have found that MNP ingestion may have detrimental effects on intestinal microbiota composition [234,235,236] and immunity [234,237,238] by inducing runaway inflammation [237,239]. Furthermore, a seminal study has potentially established a link between MNPs and the exacerbation of inflammatory bowel disease [239]. Other organ systems are similarly affected, with the liver exhibiting aberrant lipidomic profiles [240,241] and impaired lipid metabolism [242,243] while also displaying autophagy dysregulation [244] and aberrant immune activation and apoptosis [245]. The kidneys [246,247] and lungs [248,249,250] also exhibit metabolic and immune dysregulation analogous to what is seen in the intestinal system and the liver. Despite the variation in these studies of materials used, their size and dose ranges, there are broad patterns beginning to emerge which suggest that MNPs broadly affect cellular metabolism, lipid turnover and immune function. Early efforts to understand effects of MNPs on the human body also uncovered some significant challenges as to the value of the published data in terms of crafting public health policy relating to MNPs. One of the biggest challenges is estimating the amount of MNPs that are actually ingested, with the daily estimated dose being subject to a wide variety of factors such as lifestyle, geographic location, proximity to industrial activity and diet [251]. Without a reliable daily exposure dose, it is difficult to estimate safe ranges and design experiments that can produce relevant data for policy makers. In addition, effects such as weathering [252], surface chemistry [253] and the ability for MNPs to bind other potentially toxic chemicals in the environment [254] add even more confounding factors that require thorough evaluation of their toxicity before policy decisions can be taken.

These challenges are compounded when extraction and quantification of microplastics is still a hotly contested field with protocols being developed, customized and optimized based on matrix and tissue specificity [255,256,257,258,259]. Likewise, analysis techniques range wildly from cytometric to microscopic to analytical chemistry, the discussion of which is beyond the scope of this review, but a detailed discussion can be found elsewhere [260,261].

Due to these complicating factors faced by both scientists and policymakers, the prevailing thought on dealing with MNPs in terms of regulation and limitation is being contested by propositions considering remediation from the natural environment. Efforts involving physical and chemical means have been proposed to remove MNPs from the environment, particularly in the marine environment, given that the vast majority of microplastic pollution ends up in water systems. A more in-depth discussion on these methods can be found elsewhere (i.e., Pan et al., [262]). However, microbes have been found to colonize both larger plastics [263] and microplastics [264] in the ocean, with this microbial community on plastics being termed the “plastisphere” [265,266,267,268]. The detection of many hydrocarbon-degrading bacterial taxa from the orders Oceanospirallales and Alteromonadales such as *Alcanivorax*, *Marinobacter* and *Alteromonas* within the plastisphere (e.g., [269,270]) has spurred research examining whether microbes can be exploited for the purpose of degrading or recycling waterborne plastics and MNPs.

The biodegradation of plastics by microorganisms has been studied for decades (e.g., [271]), with most plastic-degrading microbes being found in terrestrial environments such as soil or plastic waste dumping sites [272]. This is likely due to the additional challenges presented by marine environments in terms of low nutrient availability and temperature [273], and marine environments have recently been found to harbour 1000-times lower polyester degrading potential than terrestrial ones [274]. While many bacteria have been reported to be capable of plastic degradation, the mechanisms by which this occurs are less frequently characterized [273]. The enzymatic degradation of plastics by bacteria typically requires a functional group within the polymer backbone, and hence, most of the described plastic-degrading enzymes to date target polyesters [272].

Probably the best-characterised plastic degradation pathway thus far is the degradation of polyethylene terephthalate (PET) by *Ideonella sakaiensis* (Proteobacteria), a bacterium that was isolated from outside a plastic bottle recycling factory and found to be capable of using PET as its major carbon source. *I. sakaiensis* degrades PET through two major steps: (i) secretion of a hydrolase, PETase, that breaks down the PET polymer to its oligomers bis- (BHET) or mono-(2-hydroxy ethyl) terephthalate (MHET); and (ii) MHET is then acted upon by a MHET hydrolase, MHETase, to produce the monomers ethylene glycol and terephthalic acid [275]. This discovery was significant because this PETase exhibited higher activity on PET than other substrates and was active at lower temperatures than previously characterised PET-degrading cutinases, laccases and hydrolases. This PETase has since been characterised [276] and engineered [277] further, and homologs with proven PET hydrolytic activity have been found in marine metagenomes [278]. Marine bacteria that can degrade PET have also been isolated, and while other Proteobacteria (e.g., *Thioclava* sp. BHET1 and *Rhodococcus pyridinivorans* P23) have been found to use the same pathway that was identified in *I. sakaiensis*, others appear to use more distinct pathways or enzymes that still require further characterisation (e.g., *Bacillus* sp. BHET2) [270,279]. The degradation intermediate ethylene glycol may be degraded using fatty acid degradation pathway enzymes [270] to acetyl-CoA, which can then enter the TCA cycle [280], while terephthalic acid may be degraded via several intermediates before entering the β-ketoadipate pathway [270].

Marine bacterial species have been found to degrade different types of plastic including PE (*Bacillus*) and PVC (polyvinyl chloride) (*Vibrio*, *Alteromonas* and *Colbetia*) [281,282]. Environmental factors such as heat and UV irradiation also play a significant role in this process by changing the surface chemistry of plastic by exposing radical groups [283], particularly for plastics such as PE and PP (polypropylene) that do not contain functional groups. These environmentally induced changes to the plastic’s chemistry and surface topology may allow for the attachment and action of cutinases, esterases and lipases that are more ubiquitous among bacterial genera and thus permit the breakdown of plastic materials. For example, an *Alcanivorax* species that is capable of degrading and growing on PE has been shown to grow more in the presence of weathered PE than pristine PE, and a range of lipases, hydrolases, and enzymes from the β-oxidation pathway for long-chain alkanes were involved in the utilisation of PE as a source of carbon and energy [284].

A point of concern is the low efficiency with which these bacteria and their respective enzymes degrade plastics; the mass of LDPE (low-density polyethylene) was reduced by 0.9% within 34 days by *Alcanivorax* and the mass of PET (polyethylene terephthalate) was reduced by 1.8% within 30 days by *Nocardioides* [284,285]. As such, efforts are currently ongoing to engineer bacteria and their enzymes for greater efficiency and speed [286,287]. Detailed analyses of enzyme efficiency, kinetics, and comparative performance of marine-derived versus terrestrial plastic-degrading enzymes under realistic oceanic conditions, including low temperature, high salinity, and nutrient limitation, have been reviewed extensively elsewhere [288,289]. However, even with increased efficiency, deploying these enzymes and bacteria in an uncontrolled manner into waterways may produce unintended consequences.

Several studies have suggested that consortia or communities may be more efficient at degrading plastics than individual isolates [290,291,292,293]. Gambarini et al. [294] recently attempted to correlate the abundance of plastic pollution with the transcription of plastic-degrading genes using predicted plastic pollution concentrations in surface waters and marine metatranscriptomes collected from various depths of the water column [13]. They found little correlation between plastic pollution levels and the abundance of transcripts for plastic-degrading enzymes, but there may be several factors playing a role in this. For example, it is difficult to gather reliable abundance data for plastic pollution. Plastics have been found to segregate across the water column according to their densities [295], and only polyethylene and polypropylene have densities lower than water. However, the presence of the plastisphere may cause buoyant plastics to sink out of surface waters [296] —where sampling has predominated to date—meaning that further sampling efforts in the deep sea are necessary. Furthermore, the use of transcription data from planktonic samples may complicate this comparison; planktonic communities and plastisphere communities are highly different [297], and it is thought that plastic-specific taxa capable of plastic degradation may only be present within the most tightly attached organisms [298].

Most bacteria appear to colonize plastics because they confer a physical support and provide advantages in resisting harsh marine environments [299]. Biofilms will develop rapidly on any surface that enters the marine environment, and organisms within these biofilms tend to be initially composed of organisms that are good at colonising surfaces and are fast-growing. These communities will go through distinct stages of ecological succession, with organisms capable of degrading the surface varying in abundance throughout [273]. It is also often suggested that the plastisphere may be more likely to be degrading the hydrophobic organic contaminants of plastics than the plastics themselves. Many plastics contain additives (up to 70% by weight) or other adsorbed pollutants that are not chemically bound to the plastics; these are often toxic and endocrine-disrupting and are able to leach out of the plastics when they encounter other hydrophobic materials. Additives that have been found in the marine environment include brominated flame retardants and plasticizers such as bisphenol A, nonylphenols and phthalates [300], and these may bioaccumulate through the food chain [301]. The ability to degrade plastic additives has been shown to be widespread, with a range of marine bacteria from the Gammaproteobacteria (*Idiomarina*, *Halomonas*, *Acinetobacter*, *Moraxella*), Alphaproteobacteria (*Novosphingobium*), Actinomycetia (*Pseudonocardia*, *Microbacterium*, *Mycobacterium*), Cytophagia (*Cyclobacterium*), and Bacilli (*Bacillus*, *Brevibacillus*) being documented to degrade plasticizers [302,303].

Biodegradation of MNPs by bacteria, their by-products or their additives may offer tantalizing new options in the efforts to remediate MNP pollution. However, these are still in their early stages of development and raise significant concerns regarding deployment and monitoring.

### 3.2. Methodologies for Further Developing Applications of Marine Bacteria

**Figure 3:**
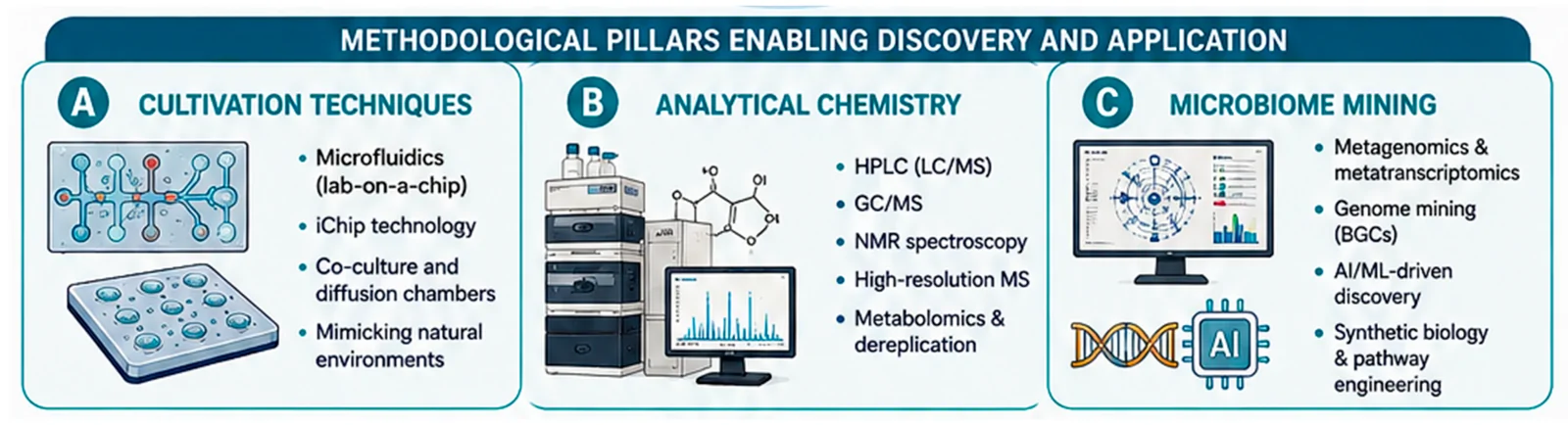
Key methodologies for advancing marine bacterial biotechnology. Marine bacterial biodiscovery is driven by three interconnected methodological pillars: (**A**) advanced cultivation techniques that expand access to previously unculturable taxa. (**B**) Analytical chemistry platforms for metabolite detection and structural characterization. (**C**) Microbiome mining (metagenomics, genome mining, bioinformatics) for rapid identification of biosynthetic gene clusters and functional potential. Integration of these approaches is essential for translating marine bacterial diversity into biotechnological applications.

Despite significant progress in leveraging marine bacteria for various applications, limitations remain in fully realizing their potential. The rapid advancements in next-generation sequencing, artificial intelligence, and high-throughput bioactivity screening platforms are poised to reshape the future of these applications across fields such as agriculture, industry, and medicine. Here, we focus on bacterial culturing, analytical chemistry, and microbiome sequencing, which underpin some of the key applications discussed earlier. It is important to note that many other methodologies are equally critical, emphasizing the need for a multidisciplinary approach to maximize the potential of these applications (Figure 3).

#### 3.2.1. Bacteria Cultivation

As noted in the review by Rotter et al. [6], the main challenge for using marine bacteria in biotechnology is the establishment of bacterial cultures. While there has been significant progress in using marine bacteria in recent years, much of the progress is related to improvements and efficiencies in omics technologies, which allows for the genetic exploration and identification of bioactivities of interest. While this information is helpful, direct access to the organism is essential for studying and developing it for biotechnology [6,304]. This section summarizes some examples of method advancements in culturing, isolating and producing marine bacteria and reviews some remaining challenges.

Marine bacteria can be difficult to culture and much of their observed phylogenetic diversity are from uncultured sources [6,305]. For decades, researchers in the field believed the dogma that these bacteria were unculturable [306]. There are many possible reasons that traditional culturing methods may not propagate marine bacteria, such as lab culture conditions failing to replicate the environmental requirements, disrupting cell-to-cell communications, and providing either insufficient or excessive substrates via the culture media that are either not usable to the marine bacteria or are at concentrations that are toxic [304]. However, since there is growing interest and need to culture marine bacteria for use in biotechnology, progress in cultivation techniques have been made.

Researchers have shown that by using different gelling agents and additives in standard culture media, the biodiversity and number of cultured marine bacteria could be improved to more closely represent the diversity reported from culture-independent methods [304]. Further, Rodrigues and de Carvalho [306] demonstrated that they could culture 45% of the bacteria from a marine pond by using several standard culture media at different dilutions (and thus nutrient concentrations) and allowing longer observation times for the development of bacterial colonies. The culturing methods used allowed for the isolation of four taxonomic orders that were not detected in the culture-independent 16S sequencing data, highlighting the importance of using both culture-dependent and -independent techniques to help characterize the microbial diversity of a habitat [306].

Advances in high-throughput culturing and screening techniques have also assisted in advancing the cultivation and selection of marine bacteria for biotechnology. For instance, to screen for exopolysaccharide production, marine bacteria were encapsulated as microdroplets, allowing for up to billions of microdroplets per mL to be screened [307], and other researchers developed a practicable high-throughput colorimetric screening assay using Congo Red agar in microplates [308]. Microdroplets coupled with the use of microfluidics can also be used to isolate and culture some previously uncultured marine bacteria, as well as screen the bacteria for bioactivities, as reviewed in Hu et al. [309]. Additionally, an isolation chip method (iChip) was coupled with fluorescence-activated cell sorting (FACS) to improve the culture recovery rate of microorganisms, demonstrating that previously unculturable bacteria can be cultured in an efficient manner [310]. Flow cytometers used for analysis and FACS have also been coupled with fluorescence in situ hybridization (FISH) to identify bacteria with biotechnological applications in bioremediation, such as hydrocarbon-degrading *Marinobacter* populations associated with eukaryotic phytoplankton in sea surface water samples [311].

While these advances have improved the cultivation of marine bacteria for biotechnology, there are remaining limitations. For example, the availability of commercial microfluidic devices is a limiting factor for widespread adoption, with few devices demonstrating success with environmental samples. Also, there are challenges around the reagents and materials required for efficient, consistent and economical microdroplet formation (e.g., high-performance surfactants and materials for microfluidic chips) [309]. Screening marine bacteria via microdroplet devices or flow cytometers is also limited by the availability of assay-specific reagents for detecting the compounds of interest, and if selecting for oil-soluble compounds there are more hurdles when using oil-based methods for microdroplet formation [307].

Advances in production, fermentation and bioprocessing technologies have improved the efficiency and scalability of producing bioactive compounds from marine bacteria. Research has focused on optimizing production processes and metabolic engineering to enhance yield and efficiency. For example, a review by García et al. [312] summarized the effect that the culture conditions have on EPS production from several marine bacteria for biotechnological extraction and use. Additionally, several genetically modified bacteria have been used to enhance the bioremediation of crude oil contamination [313] and reduce the toxicity of hazardous compounds [314]. Other strategies to enhance production of marine bacterial compounds include co-culturing with other microorganisms or applying various external stresses (e.g., chemical or environmental) to stimulate the production. For instance, co-culturing *Streptomyces* sp. 2-85 with the marine fungus *Cladosporium* sp. 3-22 led to the production of several unique antimicrobial metabolites, with notable increases in the macrolide borrelidin, along with several analogs, which can be used to combat aquatic disease in aquaculture [315]. Co-culturing has been shown to be an effective method to harness silent gene clusters to produce potential bioactive compounds for biotechnology [316,317]. Other environmental stresses, including changes to pH, temperature, salinity, carbon dioxide, or nutrient concentrations, can impact the production of bioactive compounds, such as EPSs (exopolysaccharides) where the effect in production (increase or decrease) can be species-specific and new EPS types can be generated [316]. At the industrial level, implementation of co-cultures and environmental stresses to enhanced production remains challenging, as there are still knowledge gaps regarding these complex interactions, and a systems biology analysis at the molecular level is needed. Additionally, advanced bioprocessing strategies are required to optimize and monitor the cultures to enhance production, and while advanced tools such as machine learning can facilitate these changes, it will take further research and resources to successfully implement them [318].

Overall, there have been many advances in culturing, isolating, and producing marine bacteria, but challenges remain. One major challenge is replicating the unique marine conditions required for optimal growth and production, with extremophiles presenting the most difficult challenge, as replicating the extreme environment is demanding, though possible [319,320]. The natural abundance of some marine bacterial species presents challenges, as does the unknown bacterial requirements for metabolites generated by other microbes [321]. Additionally, the genetic manipulation of marine bacteria can be convoluted due to their less characterized and unique genomes. While advances in gene editing tools, such as CRISPR-Cas systems and the lambda Red recombinase system, have helped, there is no universal system to modify any bacterium. Thus, the protocols must be tailored to the specific bacterium [322]. However, as researchers continue to test the waters and leverage innovative techniques in these areas, the potential of marine bacteria in biotechnology will continue to proliferate.

#### 3.2.2. Analytical Chemistry

Marine bacteria are well-recognized as a promising source of novel secondary metabolites with diverse biological activities, including antibacterial, antifungal, anticancer, and antiviral properties [37,155,323]. This section summarizes the screening and analytical techniques used for the identification of bioactive metabolites, polyunsaturated fatty acids (PUFAs), carotenoids, and exopolysaccharides from marine bacteria.

Marine bacteria, particularly γ-proteobacteria, are capable of biosynthesizing long-chain omega-3 PUFAs such as EPA and DHA [324,325], catalyzed by Pfa synthases [326]. Amiri-Jami et al. [327] successfully isolated a 35 kb EPA/DHA synthesis gene cluster from *Shewanella baltica* MAC1 and introduced it into food-grade lactic acid bacteria, demonstrating the potential of genetic engineering for high-value compound production. Screening for PUFA-producing bacteria has been facilitated by simplified methods such as H_2_O_2_ plate assays [328] and colorimetric TTC-based screening [329], with GC/MS serving as the primary technique for confirmation and quantification. EPA production has been extensively studied in *Shewanella electrodiphila* MAR44, achieving yields of up to 26% of total fatty acids [41]. Extraction and purification typically involve Folch lipid extraction, TLC, and silica gel column chromatography, with fatty acids analyzed by GLC or GC/MS after methylation [40,41,42,43].

Carotenoid discovery has been advanced by high-throughput screening (HTS) techniques. Asker [44] employed a 96-well plate-based HPLC-DAD-MS approach to identify 88 carotenoid-producing marine bacterial strains across five classes, including producers of astaxanthin, zeaxanthin, lutein, and canthaxanthin. Identification of novel carotenoids, such as xylosylesters, nostoxanthin, and rare C50-carotenoids, typically involves extraction with organic solvents (DMSO, acetone, or ethyl acetate), followed by silica gel chromatography, preparative HPLC, and structural elucidation via MS and NMR spectroscopy [45,46,50,52,53]. Marine bacterial exopolysaccharides (EPSs) are characterized using high-speed centrifugation for recovery, GC/MS for monosaccharide composition, FT-IR for functional group identification, and SEC or MALS for molecular weight determination, with structural analysis relying on 1D and 2D-NMR spectroscopy [54,55,56].

While HPLC, GC/MS, and NMR are powerful and well-established tools for metabolite discovery, their widespread adoption in marine microbiology laboratories is constrained by high instrument costs, substantial maintenance requirements, and the need for specialized technical expertise. These barriers are particularly prohibitive for laboratories in resource-limited settings or those in developing countries with rich marine biodiversity but limited infrastructure. To address these challenges, alternative approaches are gaining traction. Genome mining and metagenomic analysis, which use tools such as antiSMASH, PRISM, and other bioinformatics platforms, offer cost-effective means to identify biosynthetic gene clusters (BGCs) and predict metabolite structures without the need for extensive analytical instrumentation [330,331]. Additionally, recent advances in omics-based approaches are providing complementary strategies for bioremediation assessment and bioactivity screening [332]. These computational and molecular approaches, while not replacing the need for structural confirmation by analytical chemistry, can significantly reduce the reliance on expensive instrumentation during the early stages of discovery, making marine biodiscovery more accessible and cost-effective. High-throughput screening and cultivation-independent techniques continue to evolve, and their integration with affordable analytical platforms will be key to democratizing marine biodiscovery.

#### 3.2.3. Microbiome Mining

Marine microbial communities contain diverse taxa and functions that have often not yet been isolated or characterised in vitro. When mining for enzymes or taxa that are capable of certain functions, we may take a “top-down” or a “bottom-up” approach. We refer to top-down microbiome mining as starting with a microbial community and aiming to characterise it in some way to determine which taxa or functions are enriched under certain environmental conditions. These characterisations may involve taxonomic profiling through sequencing of a specific gene (for example, the 16S rRNA gene), taxonomic and functional profiling with metagenomic (MGS) or metatranscriptomic sequencing (either read-based or assembly-based), and identification of the enzymes that are produced through metaproteomics, usually followed by differential abundance testing to detect which taxa or functions are statistically significantly more abundant in sample groups of interest. We refer to bottom-up approaches as those where we start with a function or taxon of interest and screen or search for this within microbial communities. This typically involves taking a known gene or genes capable of carrying out this function and using the nucleic or amino acid sequences to search for other genes within microbial communities that have high sequence similarity.

The two main methods currently used for profiling microbial communities are marker gene and metagenomic sequencing, with marker gene sequencing typically costing approximately one sixth the cost of metagenomic sequencing. The most frequently sequenced marker gene is the 16S rRNA gene, which is ~1500 bp in length and consists of nine hypervariable regions interspersed with highly conserved regions, potentially allowing taxa to be resolved to the species level depending on the region sequenced [333]. Sequencing of one or more of the hypervariable regions has been carried out for over a decade now—and both the Earth Microbiome Project [334] and the Human Microbiome Project [335,336] recommend sequencing regions of the 16S rRNA gene (V4 or V1-V3/V4-V5, respectively)—and with the recent increases in accuracy of long-read sequencing [337,338], the full-length of the 16S rRNA gene is increasingly being sequenced. The 16S rRNA gene is most commonly used to target bacteria and archaea (although it does provide some coverage of eukaryotes), but other marker genes that are frequently utilised are the 18S rRNA gene for microbial eukaryotes and the ITS2 for fungi, along with chaperonin-60 (CPN60), ammonia monooxygenase (*amoA*/*amoB*), DNA gyrase (*gyrB*) and nitrogenase (*nifH*) genes, some of which can give information on the taxa that are contributing to particular functions [339]. Marker gene sequencing has been used extensively in marine microbiology research, allowing us to gain insights into diverse topics such as the key players within the plastisphere [297,340] and biogeochemical cycling potential [341]. The key drawback of marker gene sequencing is the limit to the functional information that it can give; however, there are several tools (e.g., PICRUSt2, Tax4Fun2 and Piphillin) that can predict the functions present within microbial communities that have been sequenced with marker gene sequencing [342,343,344]. These tools compare the sequences obtained with a database of reference genomes and use the information about the functions contained in the most similar reference genomes to predict the functions present in the microbial communities. The correlation between these predictions and the functions actually present in the communities depends on the phylogenetic conservation of the functions of interest, as well as how closely related the reference genomes are to the taxa in the sample [345], and these approaches therefore work most successfully in well-characterised environments. While sequences from marine environments often are not that similar to available reference genomes [345], there are instances where they are similar enough to be useful, as detailed below.

In metagenomic sequencing, where no specific DNA fragment is sequenced, there are typically three key analyses that are of interest: (1) taxonomic annotation of reads, (2) functional annotation of reads, and (3) assembly of reads into contigs and the subsequent binning of these contigs into Metagenome Assembled Genomes (MAGs). Taxonomic and functional annotation of reads have no minimum read depth requirement, whereas the assembly of MAGs requires sufficient sequencing depth to have good coverage of all genomic regions. There are two approaches that are typically taken for the taxonomic annotation of reads, with both approaches requiring a reference database containing genomes of known taxonomy. The first approach attempts to compare with a reference database, finding the best match or matches for each read. The second approach uses conserved marker genes that can be identified within groups of genomes in the reference database, and then the reads within the sample can be compared with these marker genes. Typically, the “all” reads approach is more sensitive (higher recall) and the marker gene approach is more specific (higher precision; [346]). There are many different tools that can be used (reviewed in [347,348,349]), but the two most widely used ones are MetaPhlAn (a marker-gene approach; [350]) and Kraken (an all reads approach; [351]). For functional annotation, reads are again compared with a database of sequences with known functions, but they may or may not be first translated to amino acid sequences; this increases the computational time to search due to the multiple combinations necessary but typically increases the accuracy of the search results. Functional annotation may be carried out with general functional databases, such as UniRef, or with specific databases for, e.g., antimicrobial resistance genes (e.g., CARD RGI), carbohydrate-metabolising genes (e.g., CAZy), virulence factors (e.g., VFDB) or phage and prophage (e.g., VIBRANT or VirSorter) [352,353,354,355,356,357]. Biological pathway information may be determined using tools such as MinPath [358], and could be supplemented with metabolomic data. While the information about taxonomy and function can be combined for read-based annotations, this is frequently only done when assembling the reads into contigs and/or the subsequent binning of these contigs and refining them into MAGs; this provides more information about the genomic context of the functions, as flanking regions of the function of interest may be present within the contigs. Many of the same methods may be used for taxonomic or functional assignment of the contigs within a MAG, but the ability to classify novel taxa or functions is much higher within MAGs than reads. MAG-based methods have now been used to collate global catalogues of marine microbial diversity, such as Ocean-M, a database containing over 50,000 MAGs with annotated biosynthetic gene clusters and antibiotic resistance genes [359], and they have also been used to identify putative novel biosynthetic gene clusters [16]. Importantly, these may allow for novel compound or enzyme discovery from uncultivated organisms, for example, from *Ca.* Eudoremicrobiaceae in Paoli et al., that were found to possess diverse biosynthetic gene clusters (BGCs) with unusual enzymology and bioactive compound structure.

After the annotation of taxa or functions of interest within samples, many analyses are often carried out, identifying variables that may be correlated with changes in the microbiome, network analyses, and differential abundance testing (reviewed in [360]). Differential abundance testing is used for identifying taxa or functions that differ statistically significantly in abundance between sample groups, and there are now many methods that are available for carrying out these tests. These tests all perform differently from one another, with varying degrees of overlap in the features detected, false discovery rate control, and sensitivity [361]. Once taxa or functions of interest have been identified, they may be cultured/isolated (see previous section) or searched for in other microbiomes using a bottom-up approach.

In many cases, a taxon or function of interest has been identified previously, and we are interested in determining whether it is present in environmental microbial communities. If we have a taxon of interest then we can use the genome of this taxon for either mapping all reads within metagenomes to determine the coverage (e.g., [270]), abundance and identity of the reads to the genome, or we may identify specific genes within this genome (i.e., the 16S rRNA gene), and we can use this to search within other available microbiomes (as we recently did with two putative plastic-degrading isolates; [270]). When a function of interest has been identified, we can use the nucleic or amino acid sequence to search for other sequences with high homology (e.g., [278]), but because the sequences of functions from different taxa often share little nucleic or amino acid sequence similarity, if multiple sequences for a function are known then these may be used to construct a Hidden Markov Model (HMM) [362]. HMMs may be constructed in nucleic or amino acid space (although are most frequently constructed in amino acid space), and they can be used to quickly screen for functions within genomes or metagenomes. For example, in the case of Polyethylene Terephthalate (PET) degradation discussed above, an enzyme (PETase) that efficiently degraded PET was identified in the soil bacterium *I. sakaiensis*. This enzyme shared significant homology with other esterases and cutinases [275], and HMMs were constructed and used to screen environmental metagenomes for other putative PET degrading enzymes. The activity of these putative PETases was confirmed experimentally, and this search allowed the phylogenetic affiliation of the enzymes to be further elucidated [278]. We were also able to use a similar HMM to identify homologs within the genomes used for functional predictions, thus predicting the abundance of PETases within marker-gene sequenced microbial communities [270].

Bioinformatic techniques may also be used to predict microbial metabolite production or abundance. These typically involve the use of genome-scale metabolic models (GEMS) for the prediction of traits like metabolite/product generation and substrate utilisation [363]; predictions come from a combination of the functional annotation of reads (as described above) with statistics and/or machine learning to map gene presence/absence to phenotypes or reaction pathways [364]. Traditionally, GEMS have been manually curated, but there are now several tools for their automated generation and prediction of metabolite changes under different conditions, (e.g., metaGEM [365] and gapseq [366]). Combining species-level GEMS with information on genome abundance within a community and potential cross-feeding/exchange can be used for predicting community-level reaction fluxes or metabolite abundances by tools like MICOM [367] or MelonnPan [368].

Many of the analyses that we have described here use command-line tools and require extensive bioinformatic expertise to carry out. There are, however, many online tools that are now available that allow: less experienced users to carry out full analysis pipelines (e.g., MicrobiomeAnalyst [369]); automated pipelines for annotating and archiving microbiome data (e.g., MGnify [370] or JGI IMG/M [371]); searching of sequences against microbial gene catalogues for finding homologues (e.g., Global Microbial Gene Catalogue [372]); the annotation of multiple sequences with general functions (e.g., BlastKOALA and GhostKOALA [373]); and the annotation of genes or genomes with specific functions (e.g., Plastic DB [374] or CARD RGI [375]).

While these tools are useful for allowing less experienced users to perform analyses without gaining years of bioinformatics expertise and they sometimes provide access to large databases that are not practical for individual researchers to download and use without large computing clusters, there are also drawbacks to their use: they typically allow for less customizability or different options for sensitivity/specificity within searches; users may be limited to performing searches with a single sequence, genome or sample at a time, which may not be practical when microbiome studies often include millions of sequences, thousands of genomes, or hundreds of samples; or the speed at which searches are able to be performed may make analyses intangible.

## 4. Conclusions

Marine bacteria harbor extraordinary metabolic and functional diversity, positioning them as a cornerstone of biotechnological innovation. Their contributions span the production of novel antimicrobials, biocontrol agents for sustainable agriculture and aquaculture, nutraceuticals for human and animal health, and bioremediation strategies for pollutants including hydrocarbons and microplastics. These applications collectively address interconnected challenges across human, animal, and environmental health, reinforcing the relevance of marine biotechnology within the One Health framework.

Despite this promise, most marine bacterial resources remain underexplored, and significant methodological bottlenecks must be overcome to translate potential into practice. Over the next five years, the field must prioritize three critical challenges: (i) improving the culturability of marine bacteria, as the majority of phylogenetic diversity remains inaccessible through traditional methods; (ii) developing robust high-throughput platforms for functional screening that bridge the gap between genomic predictions and validated bioactivities; and (iii) establishing scalable fermentation and bioprocessing systems capable of producing marine-derived compounds at industrially relevant yields. Addressing these bottlenecks will require not only continued investment in foundational research but also the integration of emerging technologies, including artificial intelligence-driven genome mining, microfluidics for single-cell cultivation, and synthetic biology tools for heterologous expression of silent biosynthetic gene clusters.

Achieving these goals demands targeted interdisciplinary collaboration. The most urgent approaches include (i) close integration of bioinformatics and analytical chemistry to accelerate compound discovery and characterization; (ii) partnerships between microbiologists and bioprocess engineers to optimize cultivation and production at scale; (iii) alignment of ecologists and environmental scientists to assess the safety and ecological impacts of deploying marine bacteria or their products in natural systems; and (iv) engagement with policymakers and industry stakeholders to navigate regulatory frameworks and ensure equitable access to marine genetic resources. These interdisciplinary efforts, grounded in the principles of the One Health framework, will be essential for unlocking the full potential of marine bacteria and delivering transformative benefits to human, animal, and environmental health as we progress through the Decade of Ocean Science for Sustainable Development.

## Figures and Tables

**Table 1:** List of carotenoids, polyunsaturated fatty acids (PUFAs), polysaccharides and other possible nutraceutical candidates reported from marine bacteria.

Bioactive Components	Source	References
Polyunsaturated fatty acids (PUFAs)		
Eicosapentaenoic acid (EPA)	*Shewanella putrefaciens* *Shewanella electrodiphila* MAR441 *Vibrio* sp.	[40,41,42]
Docosahexaenoic acid (DHA)	*Colwellia demingiae* *C. hornerae* *C. rossensis*	[43]
Carotenoids		
Astaxanthin, zeaxanthin, lutein and canthaxanthin	Flavobacteriia α-Proteobacteria γ-Proteobacteria Actinobacteria Bacilli	[44]
Diapolycopenedioc acid xylosylesters A–C	*Rubritalea squalenifaciens*	[45]
Methyl 5-glucosyl-5,6-dihydro-apo-4,4′-lycopenoate	*Planococcus maritimus* strain iso-3	[45]
Bisanhydrobacterioruberin, Trisanhydrobacterioruberin and 3,4,3′,4′-Tetrahydrospirilloxanthin	*Kocuria* sp. RAM1	[46]
Astaxanthin	*Brevundimonas* sp. strain N-5	[47]
Zeaxanthin (3R,3R)-zeaxanthin Zeaxanthin derivatives	bacterial strain GF1 *Muricauda* strain YUAB-SO-11 *Muricauda* YUAB-SO-45 Flavobacteriaceae strain YM6-073 Flavobacteriaceae strain 04OKA-17-12 *Erythrobacter* sp. SDW2	[48,49,50,51]
(3R)-saproxanthin	Flavobacteriaceae strain 04OKA-13-27	[50]
(3R,2S)-myxol	Flavobacteriaceae strain YM6-073	[50]
Nostoxanthin	*Sphingomonas* sp.	[52]
2,2′-dihydroxy-astaxanthin	*Brevundimonas scallop*	[53]
Polysaccharides		
ETW1 and ETW2	*Edwardsiella tarda*	[54]
Exopolysaccharides	*Alteromonas* spp.	[55]
EPS-DR3A	*Pseudoalteromonas* sp.	[56]
Other nutraceutical candidates		
Coenzyme Q10	*Erythrobacter*, *Sphingomonas*, *Exiguobacterium*, *Lutibacterium*, and *Bacillus*	[57]

**Table 2:** Marine biocontrol agents and the pathogens that they control.

Biocontrol Agent(s)	Pathogen(s)	References
*Alteromonas* sp. BS	*Vibrio anguillarum*, *V. damsela*, *V. splendidus*, *Pasterella piscicida*, *Pseudomonas doudorofii*	[80]
*Staphylococcus*	*Cladosporium*, *Paraphaeosphaeria*, *Trichoderma*, *Alterneria*, *Phoma*, *Arthrinium*	[81]
*Pseudoalteromonas* sp.	*Vibrio alginolyticus*, *V. anguillarum*, *V. fluvialis*, *V. harveyi*, *V. metschnikovii*, *V. splendidus*, *V. ordalii*, *V. parahaemolyticus*, *V. vulnificus* (all fish pathogens)	[82]
*Bacillus methylotrophicus*	Multiple uncharacterized bacteria	[83]
*Pseudomonas aeruginosa*	*Phytophthora infestans*	[84]
*Streptomyces*, *Micromonospora*, *Gorgonia*	*Burkholderia glumae*, *B. gladioli*, *B. plantarii*, *Fusarium oxysporum*, *Colletotrichum gloeosporioides*	[85]
*Stenotrophomonas maltophilia*	*Colleotrichum* sp., *Rhizoctonia solani*, *Curvularia* sp., *Diploidia* sp., *Fusarium* oxysporum, *Aspergillus niger*	[86]
*Bacillus* sp. PPM3	*Aspergillus flavus*, *Fusarium graminearum*, *Mucor* sp. and *Alternaria* sp.	[87]
*Paracoccus* sp.	*Prorocentrum donghaiense*	[88]
*Pseudomonas aeruginosa*	*Vibrio parahaemolyticus*	[89]
*Vibrio* sp., *Pseudomonas* sp.	*Vibrio anguillarum*, *V. splendidus*, *V. parahaemolytcus*	[90]
*Stenotrophomonas rhizophila*; *Bacillus subtilis*, *B. amyloliquefaciens*; *Pseudomonas* sp., *Cryptococcus laurentii*	*Pythium ultimum*, pepper plants	[91]
*Strombidium* sp. (a marine ciliate)	*Escherichia coli*, *Vibrio campbellii*, *Vibrio harveyi*, *Erwinia* sp., *Kluyvera* sp.	[92]
*Paenibacillus* sp.	*Fusarium oxysporum*	[93]
*Tetraselmus* sp., *Pseudoalteromonas peptidolytica*	*Vibrio parahaemolyticus*	[94]
*Bacillus subtilis*	*Magnaporthe grisea*, *Fusarium oxysporum*, *Botrytis cinerea*, *Bacillus anthracis*, *Botrytis cinerea*	[95]
*Bacillus* spp.	*Vibrio parahaemolyticus*	[96]

**Table 5:** Some marine bacteria able to degrade crude oil and some of its components.

Marine Bacteria	Contaminant(s)	References
*Rhodococcus* sp.	Crude oil	[200]
*Vibrio* sp., *Idiomarina* sp., *Kangiella* sp., *Marinobacter* sp., *Halomonas* sp.	Crude oil	[201]
*Thalassolituus* and *Oleispira*	Crude oil	[202]
*Alcanivorax*, *Erythrobacter*, *Alteromonas*, *Pseudohongiella*	Crude oil	[203]
*Luteibacter*, *Parvibaculum*, *Acinetobacter dijkshoorniae*, *Kluyvera cryocrescens*, *Acinetobacter pittii*	Crude oil	[204]
*Pseudomonas aeruginosa*, *Rhodococcus erythropolis*	Crude oil	[205]
*Cupriavidus* sp.	Crude oil	[206]
*Pseudomonas aeruginosa*	Crude oil	[207]
*Acinetobacter*, *Marinococcus*, *Methylobacterium*, *Micrococcus*, *Nocardia*, *Planococcus*, *Rhodococcus*	Crude oil	[208]
*Nesiotobacter exalbescens*	Crude oil	[209]
*Flavobacterium psychrolimnae*, *Flavobacterium petrolei* sp. nov.	Diesel	[210]
*Achromobacter* sp.	Diesel	[211]
*Rhodococcus*	Petroleum components	[212]
*Alcanivorax*, *Marinobacter*, *Pseudomonas*, *Gordonia*, *Halomonas*, *Erythrobacter*, *Brevibacterium*, *Xanthomarina*	Hydrocarbons	[213]
Gammaproteobacteria, *Acinetobacter*	Hydrocarbons	[214]
*Staphylococcus* sp.	Hydrocarbons	[215]
*Pseudomonas stutzeri*, *Bacillus simplex Bacillus pumilus*	Aromatic hydrocarbons	[216]
*Gordonia amicalis*	Alkanes	[217]
*Ochrobactrum anthropic*, *Pseudomonas mendocina*, *Microbacterium esteraromaticum*, *Pseudomonas aeruginosa*, *Stenotrophomonas maltophilia*	Polyaromatic hydrocarbons (PAH)	[218]
*Cycloclasticus spirillensus*, *Lutibacterium anuloederans*, *Neptunomonas naphthovorans*	PAH	[219,220]
Proteobacteria, *Marinobacter* and *Desulfovibrio*	Jet fuel	[221]
*Pseudomonas fluorescens*	Cd, Cu, Zn	[222]
*Vibrio harveyi*	Cd	[223]
*Enterobacter cloaceae*	Cd, Cu, Co	[224]
*Pseudomonas aeruginosa*	Cd, Hg	[225]
*Streptomyces* sp.	As	[226]

## Data Availability

No new data were created or analyzed in this study. Data sharing is not applicable to this article.
